# Remote cognitive assessment in severe mental illness: a scoping review

**DOI:** 10.1038/s41537-022-00219-x

**Published:** 2022-03-05

**Authors:** Katie M. Lavigne, Geneviève Sauvé, Delphine Raucher-Chéné, Synthia Guimond, Tania Lecomte, Christopher R. Bowie, Mahesh Menon, Shalini Lal, Todd S. Woodward, Michael D. Bodnar, Martin Lepage

**Affiliations:** 1 Department of psychiatry, McGill University, Montreal, QC Canada; 2 Department of psychology, University of Quebec in Montreal, Montreal, QC Canada; 3 Department of psychiatry, University Hospital of Reims, EPSM Marne, Reims, France; 4 Cognition, Health, and Society Laboratory (EA 6291), University of Reims Champagne-Ardenne, Reims, France; 5 Department of psychiatry, University of Ottawa, The Royal’s Institute of Mental Health Research, Ottawa, ON Canada; 6 Department of psychoeducation and psychology, University of Quebec in Outaouais, Gatineau, QC Canada; 7 Department of psychology, University of Montreal, Montreal, QC Canada; 8 Department of psychology, Queen’s University, Kingston, ON Canada; 9 Department of psychiatry, University of British Columbia, Vancouver, BC Canada; 10 School of Rehabilitation, University of Montreal, Montreal, QC Canada

**Keywords:** Schizophrenia, Human behaviour

## Abstract

Many individuals living with severe mental illness, such as schizophrenia, present cognitive deficits and reasoning biases negatively impacting clinical and functional trajectories. Remote cognitive assessment presents many opportunities for advancing research and treatment but has yet to be widely used in psychiatric populations. We conducted a scoping review of remote cognitive assessment in severe mental illness to provide an overview of available measures and guide best practices. Overall, 34 studies (*n* = 20,813 clinical participants) were reviewed and remote measures, psychometrics, facilitators, barriers, and future directions were synthesized using a logic model. We identified 82 measures assessing cognition in severe mental illness across 11 cognitive domains and four device platforms. Remote measures were generally comparable to traditional versions, though psychometric properties were infrequently reported. Facilitators included standardized procedures and wider recruitment, whereas barriers included imprecise measure adaptations, technology inaccessibility, low patient engagement, and poor digital literacy. Our review identified several remote cognitive measures in psychiatry across all cognitive domains. However, there is a need for more rigorous validation of these measures and consideration of potentially influential factors, such as sex and gender. We provide recommendations for conducting remote cognitive assessment in psychiatry and fostering high-quality research using digital technologies.

## Introduction

Cognitive impairment is a core feature of psychiatric illness, particularly schizophrenia and related disorders^1,2^. Robust cognitive deficits are observed in several cognitive domains in schizophrenia, including memory, attention, and executive function^3–5^. Less well-known cognitive symptoms in schizophrenia are cognitive biases, which are errors in judgment or interpretation that affect decision-making (e.g., jumping to conclusions, confirmation bias) and contribute to symptoms^6–8^. Both traditional cognitive impairments and elevated cognitive biases are rooted in neurobiology^9,10^ and affect many diagnosed with mental illness^11–13^, negatively impacting clinical and functional trajectories^6,14^. Cognitive assessments are essential in guiding treatment planning and, thus, proper measurement of both cognitive capacity and cognitive biases is fundamental to improve overall patient cognitive health and outcomes.

Remote cognitive assessments outside the clinic or laboratory have become a necessity in the context of the COVID-19 pandemic, which has hindered mental health initiatives in both research and clinical settings worldwide^15,16^. Yet, it also provides a rare opportunity for researchers and clinicians to draw from—and contribute to—the growing literature on remote digital technologies in psychiatry. Digital technology promoting mental health research and practice, or e-mental health, has become prevalent worldwide and can improve the implementation of evidence-based practice^17,18^. Most individuals with schizophrenia^19^ and first-episode psychosis^20^ have access to a computer, smartphone, or tablet and growing research supports the use, acceptability, feasibility, and efficacy of digital technologies in psychiatry^21–24^. Digital cognitive assessments are also being increasingly developed for these devices, with recent reviews suggesting they are feasible and reliable measures of cognition^25–27^.

Remote cognitive assessments provide many opportunities to advance research and treatment in severe mental illness, particularly schizophrenia-spectrum disorders. As they are typically digital measures, remote assessments can benefit from advances in the field of computerized neuropsychological assessment (e.g., ref. ^28^) as evidenced more broadly by the InterOrganizational Practice Committee guidelines for teleneuropsychology^29^. Remote assessments also offer the same advantages as computerized measures, including increased precision, standardized testing, and automated scoring^25,30,31^. Moreover, they enable the recruitment of larger and more diverse samples (e.g., from rural and remote areas) and of individuals who might have practical (e.g., cost, transportation) or symptomatic (e.g., social avoidance, paranoia) issues that make in-person attendance difficult. Assessments using tablets and smartphones have added benefits in that they can more easily be completed remotely at any time and in any geographic location^25,32^ and can provide data on additional dynamic variables (e.g., environment data, sleep quality, mood, level of exercise, etc.) for a broader assessment of cognition^25^.

There is an urgent need to verify that remote cognitive assessments provide valid assessments of cognitive capacity and cognitive biases in severe mental illness. Although recent reviews support the use of digital cognitive assessments in psychiatric populations, delivery in remote settings is not yet common^25–27^. Consequently, many researchers and clinicians are rapidly embarking on this path with little empirical evidence to provide guidance. The purpose of this scoping review is to provide an overview of the literature on remote cognitive assessment in severe mental illness. We focus on remote assessments in psychiatric illnesses rather than broad digital measures or remote measures in the general population given the great potential for remote assessments to drive research and treatment in this population^25,26^. We opted for a scoping review as they are designed to address broad, overarching research questions within a systematic review framework^33,34^. Our main population of interest included individuals with severe mental illness (e.g., schizophrenia-spectrum disorders), though we did not exclude research involving other groups. Our objectives were to map the current literature, identify potential barriers and facilitators, and highlight knowledge gaps in remote cognitive assessment in severe mental illness. This review aims to provide insight into the currently available options for clinicians and researchers and encourage high-quality research on remote cognitive assessment in psychiatry during and beyond the COVID-19 pandemic.

## Results

### Selection of sources of evidence

**Fig. 1:**
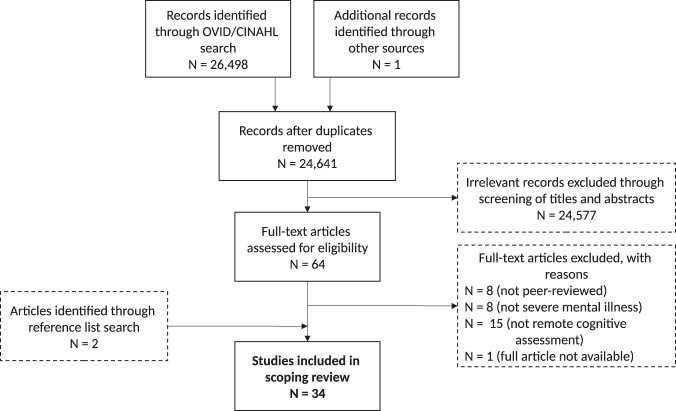
PRISMA flow diagram of article selection and reasons for exclusion. Numbers from the three searches (May 11, 2020, November 11, 2020, and February 4, 2021) are combined in this figure but described separately in the main text. *N* number of articles.

Figure 1 displays the PRISMA flowchart, combining the retrieved articles across the three literature searches. In the initial search, 24,516 references were identified, including one in press manuscript through a co-author (SG). After the removal of 1760 duplicates, titles and abstracts of 22,756 articles were randomly divided and screened by five reviewers. Of these, 57 articles were flagged as potentially relevant and full texts were screened. Upon full-text review, 32 additional articles were excluded due to not meeting one or more of the selection criteria. One additional article was identified through a reference list search. An updated search after 6 months yielded an additional 859 articles, five of which met inclusion criteria, with one additional article found through reference list search. A final updated search 3 months later yielded an additional 1124 articles (note: search updates were limited by year and overlapped with previous searches), two of which met inclusion criteria. Thus, 34 articles were included in the scoping review, including a narrative review of digital technology for remote cognitive assessment in psychiatry^26^, a commentary on remote digital cognitive assessment in schizophrenia^25^, and a systematic review on digital assessment of verbal memory in first-episode psychosis^27^. These three nonexperimental articles are incorporated only into the facilitators, barriers, and future directions sections of the logic model and the remaining articles informed all sections of the model.

### Inter- and Intra-rater reliability

Inter-rater reliability (IRR) was high at start, midpoint and end of article selection and increased over time: IRR 1 = 0.95 (SE = 0.02, 95% CI = [0.92–0.98], *p* < 0.001, two-sided); IRR 2 = 0.97 (SE = 0.01, 95% CI = [0.94–1.00], *p* < 0.001, two-sided); IRR 3 = 0.98 (SE = 0.01, 95% CI = [0.96–1.00], *p* < 0.001, two-sided). Supplementary Table 2 displays the distribution of disagreements (initial rating compared to consensus) per rater over the three IRRs. The number of articles (out of 100 at each timepoint) with conflicting ratings between two or more raters was low and decreased over time: IRR 1 = 10/100, IRR 2 = 6/100, IRR 3 = 4/100. The mean number of conflicts was also low and decreased (IRR 1 = 3.20, SD = 2.59; IRR 2 = 2.60, SD = 1.52; IRR 3 = 1.40, SD = 1.67).

### Characteristics and results of sources of evidence

Table 1 lists the 31 experimental articles selected for review (excluding the three review articles of the total selected 34 articles), along with primary characteristics (psychiatric diagnosis, sample size, remote platform, supervision, battery/measure assessed, and relevant cognitive domain). Full study characteristics are displayed in Supplementary Data, including sociodemographics (sample size, control group, age ranges, sex ratios, country, language), measure characteristics (study setting, researcher presence and title, license type, measure type, duration, alternate forms), psychometric properties, and sex-related findings. Selected articles were published between 2009 and 2021, though most (82.35%) were published within the past 5 years.

**Table 1:** Primary characteristics for selected articles.

Article	Psychiatric group (*N*)	*N*	Platform	Supervision	Battery	Measure	Domain
Atkins et al. (2017)^39^	Schizophrenia	48	Tablet	In-person	Brief assessment of cognition	Verbal memory Digit sequencing Verbal fluency Symbol coding Token motor task Tower of London Composite Score Modified Composite Score	VM WM VF SP SP REAS&EF
Bernardo-Ramos et al. (2012)^48^	Schizophrenia	30	Videoconference	Remote	Screen for cognitive impairment in psychiatry	Word learning Repetition of consonants Verbal fluency Delayed learning Visuomotor tracking Composite Score	VM WM VF VM SP
Biagianti et al. (2019)^36^	Psychosis NOS (2) Schizoaffective (16) Schizophreniform (4) Schizophrenia (82)	104	Web browser	None	Online neurocognitive assessments	Sound sweeps Visual sweeps Sustained auditory attention Sustained visual attention Auditory task switcher Visual task switcher Auditory associates Visual associates Voice choice Emotion motion Partial Composite Score	SP SP ATT ATT REAS&EF REAS&EF VM VisM SC SC
Biagianti et al.(2016)^85^	Bipolar w/ psychosis (3) Schizoaffective (15) Schizophrenia (9)	27	Tablet	None	BrainHQ-Research	Prosody Identification Task Bell-Lysaker Emotion Recognition Test	SC SC
Depp et al.(2021)^86^	Schizoaffective (35) Schizophrenia (34) Bipolar w/ psychosis (15) Depression w/ psychosis (2)	86	Smartphone	Remote	Unspecified web-based Smartphone Capable Application	Mobile Face Emotion Task	SC
Domen et al. (2019)^35^	Depressive disorder (15) OCD (36) Schizophrenia/Schizoaffective (36)	87	Web browser Tablet	In-person	My cognition quotient	Simple Reaction Time Choice Reaction Time Go no-go reaction time Verbal memory recognition Visual memory recognition N-back 1 N-back 2 Coding Trail-Making test A Trail-Making test B Composite Score Modified Composite Score	SP ATT REAS&EF VM VisM WM WM SP SP REAS&EF
Dupuy et al. (2018)^87^	Schizophrenia	22	Smartphone	In-person	Unspecified Android Application	Stroop color-word interference Letter-word generation	REAS&EF VF
Eraydin et al. (2019)^43^	Depression	7344	Web browser	None	Cambridge Brain Sciences	Verbal reasoning test Digit span task Paired associate learning task Self‐ordered search test	REAS&EF WM VisM WM
Hays et al. (2020)^51^	Schizophrenia	42	Smartphone	None	mindLAMP	Jewels trail A Jewels trail B	SP REAS&EF
Holmlund et al.(2020)^88^	Bipolar disorder (1) Major depressive disorder (8) Schizophrenia (16)	25	Smart device	N/R	Unspecified iOS software	Text recall	VM
Hung et al. (2016)^46^	Depression	54	Smartphone	None	iHOPE	Stroop Trail-making test A Trail-making test B Composite Score	REAS&EF SP REAS&EF
Kuhn et al. (2018)^89^	Depression Dysthymia	21	Web browser	None	Inquisit	Corsi block-tapping task Digit symbol substitution task Manikin test of spatial orientation and transformation Spatial reasoning task Trail-making test A Trail-making test B	WM SP ATT REAS&EF SP REAS&EF
Liu et al. (2019)^50^	Schizophrenia	18	Smartphone	None	mindLAMP	Jewel trail-making test A Jewel trail-making test B	SP REAS&EF
Ludtke et al. (2017)^52^	Schizoaffective (1) Schizophrenia (34)	35	Web browser	None	Questback Unipark Survey Software	Jumping to conclusions (scenario task)	CB
Metel et al. (2020)^42^	Anxiety (199) Bipolar (14) Depression (290) Eating disorder (50) OCD (35) Personality disorder (57) Substance dependence (24)	396	Web browser	None	Unspecified software	Davos Assessment of cognitive biases	CB
Miegel et al. (2019)^44^	OCD	130	Web browser	None	Questback Unipark Survey Software	Beliefs Questionnaire Obsessive beliefs Questionnaire	CB CB
Moritz et al. (2009)^90^	OCD	53	Web browser	None	OPST Software	Unrealistic optimism bias	CB
Moritz et al. (2012)^91^	Schizophrenia	36	Web browser	None	Questback unipark survey software	Truth effect	CB
Moritz et al. (2013)^92^	Bipolar w/ psychosis (3) Schizophrenia-spectrum (66)	69	Web browser	None	Questback unipark survey software	Effect of antipsychotic medication on emotion and cognition	CB
Moritz et al. (2015)^53^	Schizophrenia	70	Web browser	None	Questback unipark survey software	Jumping to conclusions (fish task)	CB
Moritz et al. (2015)^54^	Schizoaffective	60	Web browser	None	Questback unipark survey software	Jumping to conclusions (fish task) Modified auditory verbal learning and memory	CB VM
Moritz et al. (2018)^93^	OCD	50	Web browser	None	Questback unipark survey software	Go/No Go task auditory verbal learning and memory subjective scale to investigate cognition in schizophrenia	REAS&EF VM SUBJ
Moritz et al. (2020)^45^	Schizophrenia	101	Web browser	None	WiSo-Panel	Jumping to conclusions (box task)	CB
Parrish et al. (2021)^94^	Schizophrenia spectrum (98) Bipolar (70)	168	Smartphone	None	NeuroUX	Mobile variable difficulty list memory test	VM
Pop-Jordanova et al. (2018)^40^	Anxiety (20) Depression (35) Psychosis (15) Epilepsy (35)^a^ ADHD (30)^a^	135	Smartphone	None	NeuroGame	Reaction time	SP
Preiss et al. (2013)^95^	Bipolar depression	31	Web browser	None	CogniFit	Working memory Shifting Inhibition Visuomotor Vigilance Divided attention Auditory memory Span Composite score	WM REAS&EF REAS&EF ATT ATT WM REAS&EF
Rebchuk et al. (2020)^96^	Psychosis	39	Tablet	None	NIH Toolbox Cognition Battery abbreviated	Picture Vocabulary Oral reading Recognition Composite score (crystallized cognition) List sorting Working memory Picture sequence Memory Composite score (fluid cognition) Total score	IQ IQ WM VisM
Schvetz et al. (2021)^49^	Schizophrenia	26	Smartphone	In-person	mindLAMP	Jewels trails tests A Jewels trails tests B	SP REAS&EF
Siddi et al. (2020)^97^	Schizophrenia (11) Schizoaffective (5) Schizophreniform (4) Unspecified psychotic disorder (15) Brief psychotic disorder (1) Delusional disorder (1) Affective disorders with psychotic symptoms (8)	45	Tablet	None	Unspecified software	Digital-Corsi block-tapping test	WM
Stain et al. (2011)^98^	Depression with psychotic features (1) Psychosis NOS (3) Schizoaffective (2) Schizophrenia (5)	11	Videoconference	Remote	None	Wechsler test of adult reading WMS-R logical memory WAIS-III digit span Controlled oral word association test	IQ VM WM VF
Sumner et al. (2017)^41^	PTSD	11450	Web browser	None	Cogstate Brief Battery	Detection task Identification task Nback Visual learning	ATT SP WM VisM

*ADHD* attention-deficit hyperactivity disorder, *ATT* attention and vigilance, *CB* cognitive bias, *IQ* intelligence quotient, *NOS* not otherwise specified, *N/R* not reported, *OCD* obsessive-compulsive disorder, *PTSD* post-traumatic stress disorder, *REAS & EF* reasoning and executive function, *SC* social cognition, *SP* speed of processing, *SUBJ* subjective cognition, *VF* verbal fluency, *VM* verbal memory, *VisM* visual memory, *WM* working memory.

^a^Non-psychiatric group combined with a psychiatric group.

### Synthesis of results: logic model

**Fig. 2:**
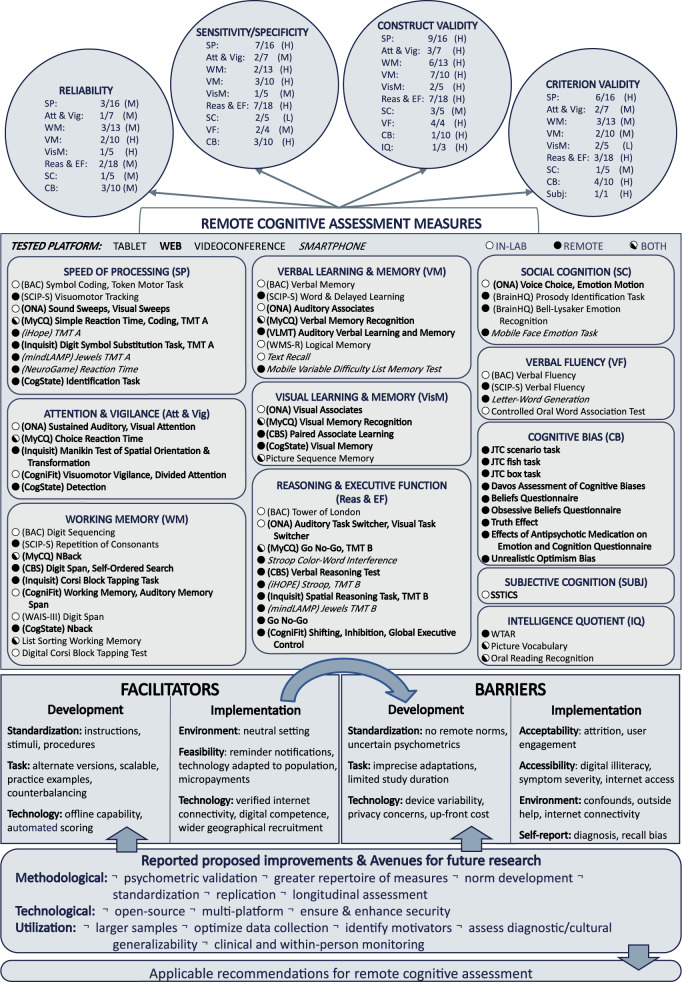
Final logic model of remote cognitive assessment measures in severe mental illness. Middle panel lists remote cognitive measures, tested platform (tablet, web, videoconference, and/or smartphone), and study type (remote, in-lab, or both) per cognitive domain. Upper circles represent the number of measures per cognitive domain in which psychometric properties (reliability, sensitivity/specificity, construct/criterion validity) were assessed over the number of measures assessing that domain. Adjacent letters summarize psychometric properties (low, L, moderate, M, high, H) detailed in Supplementary Data. Lower panels summarize facilitators, barriers, and avenues for future research, which are meant to guide future remote cognitive assessment.

The final logic model is presented in Fig. 2. The central panel includes 82 remote cognitive measures divided into 11 cognitive domains. The most assessed domains were speed of processing, working memory, reasoning, and executive function, whereas subjective cognition included only a single reviewed measure. For each measure, we illustrate which platform(s) were used (videoconference, web browser, tablet, and smartphone, in normal, bold, underline, and italic font, respectively) and whether the assessment was tested in a laboratory setting (white circle), remotely (black circle) or both (white and black circle). Briefly, two studies tested their measures using videoconferencing, 16 via web browser, two with a tablet, and nine with smartphones. Only one study^35^ reported their remote assessment could be performed on two platforms (i.e., tablet and web browser) through several used web-based measures that could likely be used on several platforms (e.g., web, smartphone, tablet). In total, six studies included remote measures that were completed in a laboratory setting, 23 were done remotely, and two used both settings.

The upper circles of the logic model summarize reported reliability, sensitivity/specificity, construct validity, and criterion validity of the reviewed measures, detailed in Supplementary Data. For each cognitive domain, we report the number of times a given psychometric was evaluated over the total number of times it was measured across studies. Next to each total, we summarize the reported psychometric properties as either low (L), moderate (M), or high (H) and invite the reader to consult Supplementary Data for detailed findings. Reliability includes estimates of internal consistency, test-retest evaluations, and intraclass correlations. Sensitivity and specificity respectively refer to the ability of the reviewed measure to identify those with and without impairments. Construct validity includes correlations with comparison measures (e.g., pen-and-paper versions) and correlations between human and automated scoring. Criterion validity includes correlations between the reviewed measures and outcomes, such as sociodemographics, symptoms, and functioning. Construct validity was most frequently assessed irrespective of the cognitive domain, whereas reliability was assessed least frequently. Overall, we observe that, for measures in which psychometric properties are assessed, remote measures were generally as reliable, sensitive, and valid as traditional measures. One exception was social cognition, which showed poor discriminatory power in one study^36^ and low to moderate correlations with traditional measures (see Supplementary Data).

The lower panels of the logic model outline thematically defined barriers and facilitators to the development and implementation of remote cognitive assessment as well as proposed improvements and avenues for future research. For development, facilitators included incorporating standardized procedures, alternate measure versions, and using technology to mitigate potential barriers (e.g., preloading stimuli to limit internet connectivity issues). On the other hand, developmental barriers included confidentiality concerns, technology/system variability, imprecise measure adaptations, and the current lack of remote norms. For implementation, testing in a neutral setting, improving feasibility (reminders, user-friendly technology), and wider access to individuals living in rural regions have been identified as facilitators. Inversely, low participant engagement, symptom severity, limited digital literacy, poor technology accessibility, and potential access to outside help (e.g., through family members or the internet) have been identified as barriers. As for proposed improvements and future directions, the authors of reviewed studies highlighted the need for further psychometric validation, development of remote norms, and strategies to ensure digital security. There were also proposed improvements pertaining to the promotion of open-source options, optimization of collected data (detailed cognitive performance data and additional contextual variables, such as sleep and physical activity), and verification of diagnostic and cultural generalizability.

### Sex and gender considerations

Given the well-documented sex differences in cognition and their relevance to psychiatric illness^37,38^, we sought to examine the role of sex and gender on remote assessment of cognitive capacity and cognitive biases. Approximately one-quarter of experimental studies (*n* = 9) reported on differences based on sex assigned at birth (male, female) and none on gender identity (e.g., non-binary, trans-, cis-, genderfluid). Sex and gender were often used interchangeably presumably in reference to sex assigned at birth. One study reported matching participants based on sex and used sex-corrected pen-and-paper norms^39^, one did not report explicit sex ratios^40^, and one included females only^41^. Those that reported on sex differences found that females displayed higher cognitive biases^42^ and lower performance on working memory^43^. Two articles described nonspecific sex differences^36,44^, and three found no sex-related performance^40,45^ or attrition^46^ differences (see Supplementary Data).

## Discussion

The present study provides a scoping review of the literature on remote assessment of cognitive capacity and cognitive biases in severe mental illness to map current knowledge and inform clinicians and researchers on best practices. In total, more than 26,000 articles were retrieved and 34 met our inclusion criteria. Identified measures generally showed acceptable psychometric properties, though these were assessed in less than half of reviewed studies. Facilitators and barriers to the development and implementation of remote cognitive assessment measures, as well as future research directions proposed by identified studies, provide clear considerations for future research and practice. This work brings together the current library of remote cognitive measures in psychiatry that researchers and clinicians may consult based on their needs, including cognitive domain, remote platform, and level of supervision required. Below we provide general recommendations and considerations to foster remote cognitive assessment in psychiatry.

Our scoping review did not identify a “gold-standard” remote battery for a comprehensive assessment of cognition in psychiatric populations. Moreover, there is currently no single cognitive battery, remote or otherwise, assessing both cognitive capacity and cognitive biases to provide an overall measure of cognitive health in severe mental illness. For cognitive capacity, the two most frequently used computerized cognitive batteries in psychiatric populations (CANTAB and CogState)^47^ did not emerge strongly in our review, suggesting they have not yet been adopted extensively in remote settings despite their potential for remote administration. Only one study^41^ used the CogState Brief Battery in a remote setting in a very large sample of nurses with elevated PTSD symptoms, though the generalizability of the results to other psychiatric samples remains in question. CANTAB was only used in a single study as an in-lab comparison measure^35^. Moreover, social cognition measures were restricted to emotion recognition tasks and tests of other domains of social cognition (e.g., theory of mind) are currently lacking. Notable comprehensive remote batteries that reported acceptable psychometric properties included the Brief Assessment of Cognition^39^, My Cognition Quotient^35^, Online Neurocognitive Assessments^36^, and Screen for Cognitive Assessment in Psychiatry^48^. Some individual tasks also showed valid, sensitive, and/or reliable remote administration, particularly the Jewel Trail Making Task from the mindLAMP smartphone application, used in three studies^49–51^.

Cognitive biases were primarily assessed using scales rather than tasks, which are more amenable to remote administration via online survey platforms. Importantly, most cognitive bias scales and all cognitive bias tasks identified were designed to address individual biases, such as jumping to conclusions^45,52–54^. The most general measure of cognitive biases identified was the Davos Assessment of Cognitive Biases Scale^55^, though it does not measure all biases reported in psychiatric disorders. Surprisingly, the well-known Cognitive Biases Questionnaire for Psychosis^56^ did not emerge in our review, suggesting it has yet to be used in remote settings with severe mental illness. Given the importance of cognitive biases in understanding and treating the symptoms of severe mental illness^7^, the development of a validated remote cognitive bias battery to complement the numerous batteries that exist to assess cognitive capacity is recommended.

Fundamentally, the question of which measure(s) to use depends on the cognitive domain(s) of interest and other pragmatic considerations (platform, duration, cost, etc). Comprehensive batteries would likely be most convenient for clinicians and for researchers interested in general measures of cognition across various domains. However, most of the available comprehensive cognitive batteries are proprietary (Supplementary Data) and thus incur significant costs and less flexibility for the user. Several open-source measures were available through online platforms, such as Inquisit Web or researcher-developed applications. There exist other promising experiment-sharing platforms (e.g., Pavlovia, Expyriment, CognitionLab), though, to our knowledge, these have yet to be tested remotely with psychiatric samples. Generally, these platforms require “picking and choosing” and/or developing cognitive measures and thus necessitate greater reflection on the objectives and cognitive measures of interest. True open-source alternatives, in which the task’s source code is fully accessible are also available for some measures, or reportedly available from the authors. These initiatives would likely be of greater interest to cognitive scientists.

While this review illustrates that remote cognitive assessment is feasible with psychiatric populations, most studies strongly recommended further validation of existing remote measures, development of additional measures, and remote norms. Remote norms were not reported in the identified studies, despite the potential for remote studies to collect data from large and diverse samples and the growing number of computerized batteries with normative data (e.g., refs. ^57–60^). Only one selected study assessed whether in-lab computerized scores were comparable to pen-and-paper norms, finding that modifications were necessary for some subtests of the Brief Assessment of Cognition^39^. Thus, normative data derived from in-person assessments might not be applicable to computerized or remote versions of all cognitive tests. The development of remote norms would greatly facilitate remote cognitive assessment and allow for improved comparisons between studies. However, this poses several challenges. Notably, comparable in-person normative data are not available for all tests, particularly for measures of cognitive biases. In addition, the nature of remote assessment occurring outside the laboratory naturally reduces researchers’ control over environmental confounds that could affect test performance. Future development of remote normative data and guidelines for such norms should address these potential issues.

Additional quality considerations should be made during both the development and implementation of a new cognitive task or study. In terms of development, identified studies strongly encouraged using standardized and automated procedures, including instructions and scoring, to reduce variability and human error. Moreover, eliminating the need for a synchronous internet connection (e.g., preloading cognitive stimuli and allowing test results to be uploaded asynchronously) can mitigate potential issues with internet connectivity. Adaptation of certain pen-and-paper measures to remote computerized software also presents a major challenge to validity and feasibility, particularly for those measures that involve writing or motor skills, and pen-and-paper norms may be inaccurate in these cases. The choice of remote platform (web, tablet, smartphone, videoconference) or multi-platform options should also be carefully evaluated, as platforms vary in terms of functionality (e.g., touch screen ability) and other parameters (e.g., screen size, computational power) that can affect performance. It is also imperative to ensure that collected data corresponds to high ethical standards in terms of security and privacy, including transparency, confidentiality, data safeguarding, and avoiding superfluous data collection^61,62^. Finally, when implementing cognitive assessments in remote settings, participants’ digital competence, symptom severity, and potential environmental distractors should be considered, all of which can affect performance over and above cognitive impairments. Reminder notifications, standardized instructions, practice, and remote monitoring may limit these potential issues.

Future remote studies should prioritize larger samples, standardization of instructions and environment, where possible, broader data collection (e.g., environmental data, sleep quality, mood, level of exercise, etc.) and wider recruitment (e.g., remote and rural areas) to allow for the development of norms and to assess potential sociodemographic factors (sex, gender, race, education, etc.) and diagnostic and cultural generalizability. Development and validation of additional remote measures of both cognitive capacity and cognitive biases would also bring us closer to developing an overall battery of cognitive health for those with psychiatric disorders.

Quality remote cognitive assessments have strong implications for remote cognitive interventions in psychiatry. Effective cognitive interventions are available for both cognitive capacity (e.g., cognitive remediation therapy)^63–66^ and cognitive biases (e.g., metacognitive training, cognitive behavioral therapy for psychosis)^6,67,68^. In a complimentary review and meta-analysis on the efficacy of virtual evidence-based psychosocial interventions for schizophrenia-spectrum disorders^69^, 11 studies met inclusion criteria for virtually-delivered cognitive remediation. Six of these were included in a meta-analysis showing moderate effects on neurocognition (Hedges g = 0.35) and functioning (g = 0.33), similar to in-person interventions^66^. These initial results on efficacy are promising for virtual adaptations of existing interventions and encourage the development of new programs specifically designed for virtual delivery. For example, patient-tailored remote interventions following a preliminary remote cognitive assessment would integrate personalized treatment and broad accessibility.

The current study presents several strengths. First, it is a broad scoping review of remote measures of both cognitive capacity and cognitive biases in severe mental illness designed to address an urgent need given the COVID-19 pandemic. Second, it involves rigorous methodological procedures including randomization, repeated inter-rater reliability, extensive quality control, and iterative data synthesis. Third, the search was updated after six and nine months given the rapidly evolving literature in this domain. Finally, data extraction was comprehensive and included several characteristics (e.g., diagnosis, setting, researcher presence, platform, duration, alternate forms, licensing, cognitive domain, psychometric properties) that will assist researchers and clinicians in their choice of remote measures.

A potential limitation of this study is that the search strategy, which was focused on severe mental illness, may not have captured all articles assessing remote cognition in other psychiatric disorders, though several were identified, and reference lists were also checked. Additionally, we did not calculate quality scores for included studies. Contrary to systematic literature reviews, a critical appraisal of sources of evidence is not generally indicated for scoping reviews, which are meant to be broadly inclusive of the literature^70^. Third, despite our best efforts, our review may have missed some findings from unpublished studies and ongoing investigations. This is particularly relevant given the present surge in remote research due to the COVID-19 pandemic and is illustrated by the eight additional sources of evidence identified in our updated searches. There are also many additional remote cognitive measures and batteries that were identified during the review process, but these had not yet been tested in populations with severe mental illness and were outside the scope of this review. Lastly, our domain classifications may not accurately represent all cognitive function(s) assessed by a given measure. However, this classification was developed using an iterative process until consensus was reached by the three lead authors and was reviewed and approved by the remaining authors, all of whom are experienced in the field.

At present, researchers and clinicians in psychiatry can choose from a vast selection of remote cognitive measures assessing many cognitive domains through various remote platforms. However, there is an urgent need for more rigorous validation of these measures and for a stronger consideration of influential factors, such as sex and gender differences and cultural diversity. Remote cognitive assessment is necessary given the current climate but also has the potential to propel the field of cognitive psychiatry forward. In conclusion, this review provides clinicians and researchers with a comprehensive list of remote cognitive assessment measures as well as insight into methodological and practical considerations that may serve as a first step in the development of guidelines for remote cognitive assessment in severe mental illness.

## Methods

### Protocol and registration

The review protocol was preregistered on the Open Science Framework: https://osf.io/cbzq8 (Registration 10.17605/OSF.IO/CBZQ8) and followed the PRISMA extension for scoping reviews^71^ (see Supplementary Table 3 for PRISMA checklist) and the Joanna Briggs Institute guidance on conducting systematic scoping reviews^34,70,72^.

### Search strategy and selection criteria

A comprehensive literature search was conducted on May 11, 2020 and updated on November 11, 2020, and February 4, 2021 using OVID (MEDLINE, PsycInfo, and EMBASE) and EBSCO (CINAHL) databases. The following keywords were used: (schizophreni* OR psychosis OR psychoses OR psychotic* OR severe mental illness) AND (cogniti* OR neuropsych* OR bias* OR reason*) AND (remote* OR online* OR mobile* OR digital*) AND (assessment OR evaluat* OR test* OR measure*). The search was limited to articles in either English or French from any publication year. Evidence sources included peer-reviewed research articles, reviews, and letters to the editor, excluding books and conference abstracts. Repositories of tests and measures were searched (PsycTESTS, Health and Psychosocial Instruments, Mental Measurements Yearbook), experts were contacted for unpublished findings, and reference lists of selected articles were examined for additional studies.

### Article screening

Retrieved articles were combined in Endnote software, and in a first pass, duplicates were excluded automatically by comparing Author, Year, Title, and Journal fields. Duplicates based on all possible other combinations of these fields were produced and checked manually. The remaining articles were randomized for initial screening based on title and abstract. Due to the urgent nature of this review, five raters were assigned to screen the remaining de-duplicated articles, with each rater screening approximately one-fifth of the total number of de-duplicated articles. Raters assigned each article one of three possible ratings: include, questionable, exclude. To determine whether questionable articles should be included or excluded, full texts were reviewed according to the study inclusion criteria and a consensus was reached by the research team.

Article screening was based on the following eligibility criteria: (a) peer-reviewed; (b) included individuals with a diagnosis involving severe mental illness (e.g., schizophrenia-spectrum disorders); and (c) reported on the remote assessment of cognitive capacity and/or cognitive biases. During article selection, we recognized that several articles included a broad range of diagnostic groups (e.g., anxiety, depression, OCD) and we included these conditions to maintain a broader scope. In addition, many articles assessed remote cognitive tasks in a laboratory setting (e.g., comparison with a standard pen-and-paper battery). In order to include these articles, which were not technically remote, while not including all articles reporting on computerized cognitive assessment in psychiatry, we included these on a case-by-case basis, and the inclusion of articles determined via consensus. Selected articles were then retrieved for full-text screening and data extraction of included articles.

Given that articles were screened by different raters, rather than by all raters, we assessed inter-rater reliability (IRR) by having all raters assign ratings to three samples of 100 articles at the start, midpoint, and end of article selection, as in previous research^73^. IRR was calculated using Gwet’s AC_1_ statistic^74^ via the R AgreeStat package to account for the kappa paradox, in which unusually low agreement statistics are produced when there is a skewed distribution of ratings (e.g., many excluded articles)^75–77^ (see Supplementary Table 1 for a demonstration with the current data). Following each IRR timepoint, raters produced a consensus for any inconsistent ratings. Intra-rater reliability was also assessed across IRR timepoints^73^ by comparing each rater’s accuracy relative to consensus.

### Data extraction

Data extraction was performed on selected articles according to a pre-developed form, which was tested and fine-tuned with one exemplar article by the lead author. Articles were randomized for data extraction across three independent raters. Data extraction was quality controlled by authors K.M.L., G.S., and D.R.-C. by randomly selecting six articles (10% of articles originally extracted) and re-extracting the data. Data extraction included the following predetermined variables: bibliographic data (authors, year, title, abstract), study characteristics (aims, design, country, setting, researcher presence/title, sample size, psychiatric diagnosis, mean age, age range, sex/gender ratio), description of remote assessment methods (remote/comparison measure(s), battery, remote platform, developer, language, duration, alternate forms, availability of norms), main findings, sex/gender findings, psychometric properties (reliability, sensitivity/specificity, construct validity, criterion validity), facilitators, barriers, and future directions.

### Synthesis of results

Data were synthesized and illustrated using the logic model methodology, following the W. K. Kellogg Foundation guidelines^78^ and previous research^79,80^. This flexible method uses an iterative approach to identify and illustrate thematic categories and the putative underlying links to portray complex relationships^81,82^. In this study, the logic model was used to classify cognitive measures into domains (speed of processing, attention and vigilance, working memory, verbal learning and memory, visual learning and memory, reasoning and executive function, social cognition, verbal fluency, cognitive bias, subjective cognition, and IQ), expanded from the MATRICS^83^ classification. The logic model also outlines psychometric properties, facilitators, barriers, and future directions identified.

### Logic model development

Cognitive measures were categorized into cognitive domains, which were inspired by the MATRICS^83^ classification: speed of processing, attention and vigilance, working memory, verbal learning and memory, visual learning and memory, reasoning and problem solving, and social cognition. We added verbal fluency, cognitive bias, subjective cognition, and IQ domains, to account for identified measures which did not fit within the MATRICS domains. We initially selected the MATRICS classification as it provides a well-known framework for cognitive impairment in schizophrenia, which was our primary population of interest and the group assessed in most studies. In addition, the MATRICS domain of reasoning and problem solving was relabeled as “reasoning and executive function” in order to include additional measures of executive functioning (e.g., inhibitory control) without creating a separate domain. Notably, several measures tap into additional domains reported in the literature (e.g., visuomotor processing) or recruit additional cognitive processes that fall into other identified domains (e.g., speed of processing measures also require attention). In the current review, a given measure’s primary cognitive domain is reported and was determined through consensus.

### Differences between draft and final logic model

Prior to data extraction, we developed a draft logic model (Supplementary Fig. 1). The final logic model (Fig. 2) was developed through an iterative process by the three lead authors and was reviewed and approved by the remaining authors. Differences between the draft and final logic models are outlined below. In both models, the identified remote cognitive measures, relevant cognitive domains, and procedural characteristics are displayed in the middle panel. Psychometric properties are located in the upper circles and facilitators, barriers, and future directions in the lower sections.

The draft logic model categorized the identified remote cognitive assessment measures categorized by MATRICS cognitive domain (speed of processing, attention/vigilance, working memory, verbal memory and learning, visual memory and learning, reasoning and problem solving, social cognition)^83^ with the addition of a cognitive bias domain. The draft model also outlined utilized procedures (setting, platform, researcher presence/title, duration, material, cost), psychometric properties (reliability, sensitivity/specificity, concurrent validity, predictive validity), facilitators, barriers, improvements/future research, and recommendations. During data extraction, we decided to report on the license type (proprietary, open-source) of a given measure/battery, rather than cost, as this was not readily available. To simplify the presentation, the final logic model reports only the platform and location of testing with the other procedures relegated to Supplementary Data. We also renamed concurrent and predictive validity to construct and criterion validity, respectively, to emphasize the wider breadth of psychometric properties that were available. Facilitators, barriers, improvements/future research remained unchanged from the draft to the final logic model.

## Supplementary information

Supplementary Information

Dataset 1

## Data Availability

Data generated from this study are available via the Open Science Framework (OSF; https://osf.io/wh6vt/) with the identifier 10.17605/OSF.IO/WH6VT^84^.

## Code availability

Inter-rater reliability was calculated with the AgreeStat package in R, available at: https://agreestat.com/software/r/new/agree.coeff3.raw.r.
